# Metagenome Plasticity of the Bovine Abomasal Microbiota in Immune Animals in Response to *Ostertagia Ostertagi* Infection

**DOI:** 10.1371/journal.pone.0024417

**Published:** 2011-09-09

**Authors:** Robert W. Li, Sitao Wu, Weizhong Li, Ying Huang, Louis C. Gasbarre

**Affiliations:** 1 Animal and Natural Resources Institute, United States Department of Agriculture, Agricultural Research Service, Beltsville, Maryland, United States of America; 2 Center for Research in Biological Systems, University of California San Diego, San Diego, La Jolla, California, United States of America; Cairo University, Egypt

## Abstract

Infections in cattle by the abomasal nematode *Ostertagia ostertagi* result in impaired gastrointestinal function. Six partially immune animals were developed using multiple drug-attenuated infections, and these animals displayed reduced worm burdens and a slightly elevated abomasal pH upon reinfection. In this study, we characterized the abomasal microbiota in response to reinfection using metagenomic tools. Compared to uninfected controls, infection did not induce a significant change in the microbial community composition in immune animals. 16S rRNA gene-based phylogenetic analysis identified 15 phyla in the bovine abomasal microbiota with Bacteroidetes (60.5%), Firmicutes (27.1%), Proteobacteria (7.2%), Spirochates (2.9%), and Fibrobacteres (1.5%) being the most predominant. The number of prokaryotic genera and operational taxonomic units (OTU) identified in the abomasal microbial community was 70.8±19.8 (mean ± SD) and 90.3±2.9, respectively. However, the core microbiome comprised of 32 genera and 72 OTU. Infection seemingly had a minimal impact on the abomasal microbial diversity at a genus level in immune animals. Proteins predicted from whole genome shotgun (WGS) DNA sequences were assigned to 5,408 Pfam and 3,381 COG families, demonstrating dazzling arrays of functional diversity in bovine abomasal microbial communities. However, none of COG functional classes were significantly impacted by infection. Our results demonstrate that immune animals may develop abilities to maintain proper stability of their abomasal microbial ecosystem. A minimal disruption in the bovine abomasal microbiota by reinfection may contribute equally to the restoration of gastric function in immune animals.

## Introduction

Abomasal pH plays an important role in maintaining proper gut physiological function and serves as a potent natural barrier against infections by bacterial pathogens. Pepsin, one of three major proteolytic enzymes of host origin, is secreted by chief cells in an inactive form, pepsinogen, whose cleavage and activation require hydrochloric acid released from parietal cells in the abomasal mucosa. Once activated, pepsin needs a low pH environment (generally pH 2–3) for its proper function. Pepsin starts to denature when pH rises to about 5 and becomes permanently inactivated at pH 6 and above. Adequate gastric pH levels are necessary for protein digestion and absorption of certain ions (calcium and iron) and vitamins (B12). Abomasal pH imbalance often has important pathological and physiological consequences. Therefore, abomasal pH is under delicate physiological control. For example, gastrin is an important peptide hormone stimulating hydrochloric acid secretion and regulating pepsinogen production.

*Ostertagia ostertagi* is a major abomasal nematode in ruminants with a significant economic impact in temperate regions of the world. One of the major pathophysiological manifestations associated with ostertagiasis in cattle is impaired gastrointestinal function, including reduced gut motility and decreased gastric acid secretion [1]. During a primary infection, *Ostertagia ostertagi* induces a marked reduction in gastric acid secretion, due mainly to a loss in parietal and chief cells. As a consequence, abomasal acidity decreases from a physiologically normal pH 1 to 3 to pH 5 to 6 or higher. By 28 days post infection, terminal abomasal pH in infected animals is significantly elevated to pH 6.6 from pH 2.9 in naïve animals [2]. Serum pepsinogen levels are also significantly elevated by infection. Concurrently, hypergastrinaemia is often evident during a primary infection. In sheep, the anaerobic fraction of the abomasal microbiota expands rapidly when abomasal pH increases to 3.5 and above during a primary infection [3]. These changes directly contribute to impaired gastrointestinal function and ensuing productivity compromise. Clinical symptoms of moderate to severe *Ostertagia* infections in young calves may include diarrhea, malnutrition and even death. However, blood and gastric parameters affecting gut motility, gastrointestinal secretion, and subsequent protein metabolism and nutrient absorption have not been closely examined in immune animals, which were developed using multiple drug-attenuated infection-treatment cycles. Immune animals have a significant reduction in mean worm counts and an increase in the percentage of larvae up to reinfection. Recently, host mechanisms contributing to the development of long-term protective immunity have been identified [4]. However, parasite-induced alterations in gut microbiota remain unknown, and the bovine abomasal microbiota in response to external stresses has yet to receive sufficient attention. Alterations of the abomasal microbiota have an important consequence in cattle. Several abomasal diseases directly result from uncontrolled proliferation of bacteria [5]–[6]. In addition, parasite infections and subsequent alterations of gut microbiota have the potential to modify host nutrient requirements. Therefore, three-way interactions between the host, gut microbiota, and parasites should be closely examined in order to gain a holistic understanding of host-pathogen relationship. In this study, we attempt to characterize the bovine abomasal microbiota in immune animals in response to parasite infection to gain further insight into the mechanisms of protective immunity against *Ostertagia ostertagi* infections in cattle.

## Results

### Worm burdens, abomasal pH, sequencing and assembly characteristics

Abomasal luminal pH was measured post mortem. The luminal pH was significantly elevated in immune animals by reinfection at 14 days post infection (1.90±0.13 and 2.83±0.03 in control and infected groups, respectively; *N* = 3; *P*<0.05). However, a luminal pH 2.8 is still considered within the normal physiological range. The number of parasites recovered from the abomasum is listed in Table 1. Most notably, the percentage of larvae recovered in the immune group was significantly different at 13.2% as compared to <5.0% recovered from the primarily infected group. A low luminal pH and a retarded development of larvae were important indicators of a strong protective immunity in these immune animals. This was consistent with an earlier observation that previous infections tend to increase the proportion of larvae [7].

**Table 1:** Abomasal pH and worm burdens in immune cattle in response to *Ostertagia ostertagi* infection.

	Control	Infected
Age (day)	259.5±2.5	259.5±1.5
Abomasal pH	1.90±0.13	2.83±0.03*
Worms	10±1.0	5,855±2,875
Input sequences		
16S V3–V5	36,944±21,906.3	45,890±29,867.0
WGS	270,873±58,688.4	250,432±32,684.6
Host sequences	40,235±3,058.9	14,226.5±3,857.3
Assembly		
N50	536.5±9.2	678±110.3
Contig length (bp)	699.8±33.6	711.1±72.8
Contig#	23,594±4515.6	24,144±770.8
Assembled length, Mb	16.58±3.95	17.17±1.81

The assembly characteristics of whole genome shotgun (WGS) sequence reads are also listed. The numbers denote Mean ± SD (*N* = 3).

*P<0.05 based on unpaired *t*-test.

**Figure 1:**
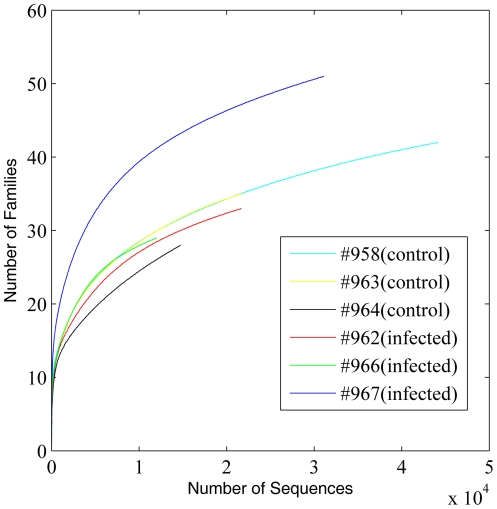
Family-level rarefaction curves of control and infected animals based on the 16S rDNA sequences analyzed using RDP Classifier with a confidence threshold of 80%.

**Figure 2:**
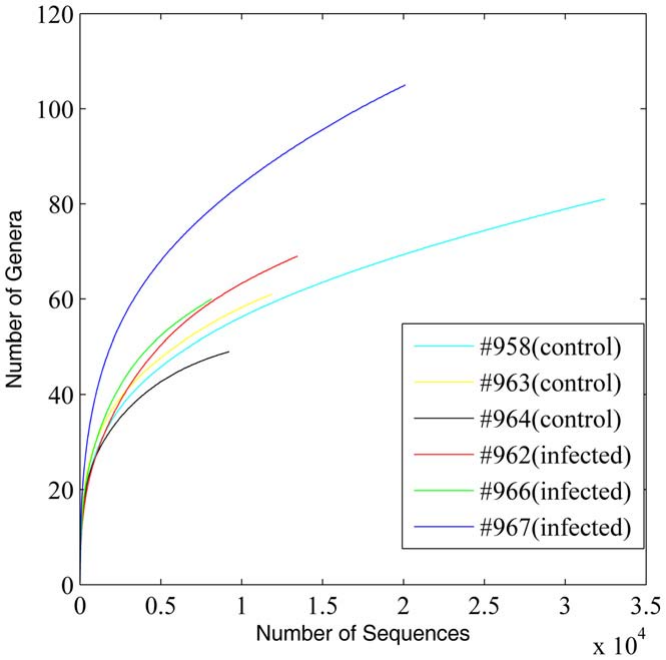
Genus-level rarefaction curves of control and infected animals based on the 16S rDNA sequences analyzed using RDP Classifier with a confidence threshold of 80%.

**Figure 3:**
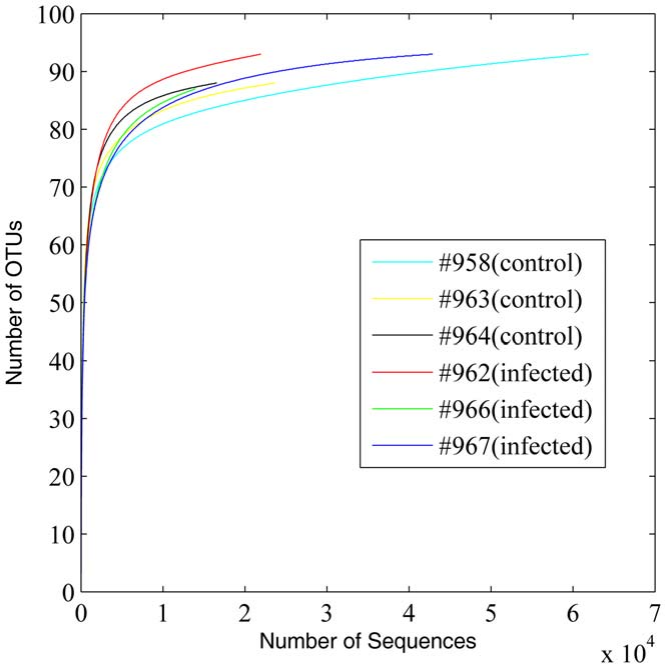
OTU-level rarefaction curves of control and infected animals based on the 16S rDNA sequences. The 16S rDNA sequences were analyzed using CD-HIT-OTU.

16S rRNA gene (rDNA) sequence reads were checked for possible artifacts and reads shorter than 200 bp were excluded for analysis. Rarefaction curve analysis was conducted at levels of family, genus, and OTU (Figures 1, 2 and 3). Species (OTU)-level rarefaction analysis suggests that our sequencing depth for this study was adequate (Figure 3). Raw sequences from the whole genome shotgun (WGS) approach (mean read length = 425 bp) and their assembly characteristics are listed in Table 1. Quality control steps, including removal of sequences of host origin, resulted in a reduction of 15.1% and 5.8% of raw reads from control and infected groups, respectively. These quality control steps resulted in retention of mean sequence reads of 230,638 and 236,205 from control and infected groups, respectively. These reads were *de novo* assembled using the Newbler software (v2.5.3). Approximately 66.9% and 61.7% of input WGS reads from control and infected groups were assembled, resulting in a total of 16.59 Mb and 17.16 Mb of assembled sequences for control and infected groups, respectively. 56,227 and 67,937 singletons were at a length of ≥300 bp. These singletons and contigs of length ≥300 bp were further functionally annotated against the Pfam, COG and KEGG databases.

### Characteristics of the bovine abomasal microbiota based on 16S sequences

**Figure 4:**
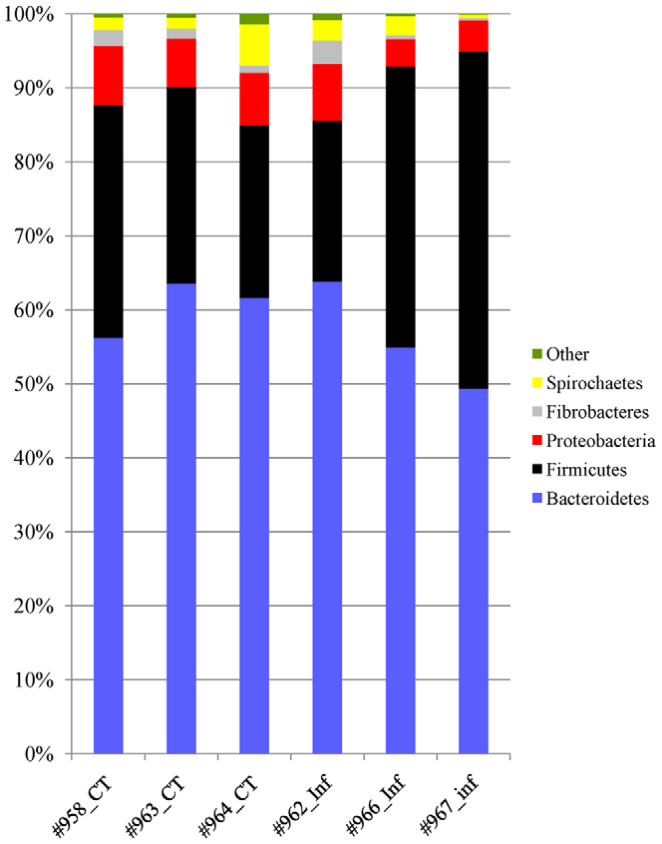
The relative distribution of the five most abundant phyla in the bovine abomasal microbiota of immune animals. The number denotes percentages of 16S sequences assigned to a given phylum using RDP Classifier at an 80% confidence threshold. CT: control; Inf: infected.

**Figure 5:**
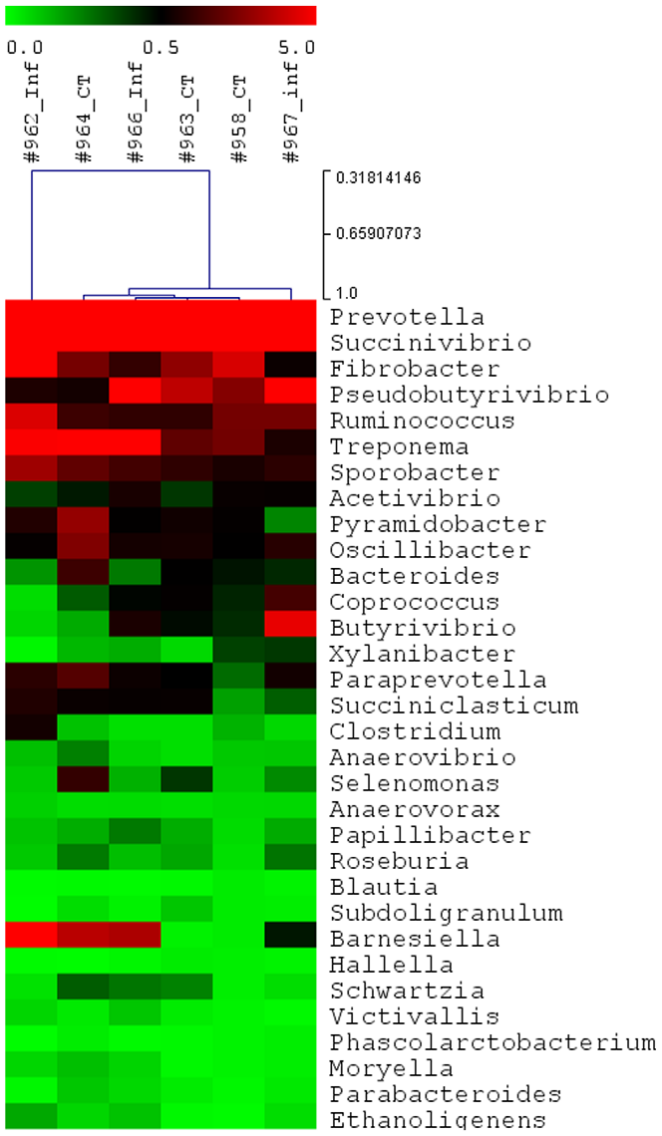
A heatmap of the bovine abomasal microbiota composition at a genus level. 32 genera that shared by all samples tested (core microbiome) were displayed. The scale (log 2) was the percentage composition based on the 16S rDNA sequences using RDP Classifier at an 80% confidence threshold.

16S rRNA gene-based phylogenetic analysis was conducted using RDP Classifier at 2 levels of confidence threshold, 80% and 95%. The numbers listed were based on a confidence threshold of 80% unless noted otherwise. Fifteen phyla from the abomasal microbial community were identified. Bacteroidetes, Firmicutes, Proteobacteria, Fibrobacteres, and Spirochates were the most abundant phyla in the bovine abomasal microbiota (Figure 4). Infection in immune animals seemed to induce insignificant changes in phylum-level composition of the abomasal microbiota compared to that of control animals. The number of families and genera in a typical abomasal microbial community was 37.0±9.5 and 70.8±19.8, respectively. The 5 most abundant families in control immune animals were *Prevotellaceae* (52.8%), followed by *Lachnospiraceae* (15.4%), *Ruminococcaceae* (11.1%), *Succinivibrionaceae* (7.1%), and *Spirochaetaceae* (4.3%). At the genus level, *Prevotella* was the most abundant, accounting for 63.1% and 46.4% of 16S rDNA sequences that were positively assigned in the microbiota of control and infected animals, respectively. *Succinivibrio* was ranked second in terms of its relative abundance, representing 10.9% and 7.9% in the abomasal microbial communities of both groups, respectively. There were 10 genera with their relative abundance ≥1.0% in the microbiota of control animals. Thirty two genera were shared by all samples tested, possibly representing the core microbiome of the bovine abomasal community (Figure 5). Infection in immune cattle seemingly had only a minor impact on genus-level abomasal microbiota composition. Five genera with a low level of abundance (<0.2%) were marginally impacted by infection (*P*<0.1 based on *t*-test). These genera included *Ethanoligenens* (mean ± SD = 0.03%±0.04% and 0.12%±0.05% in control and infected animals, respectively) and *Subdoligranulum* (0.07%±0.04% and 0.02%±0.02% in control and infected animals, respectively), two of 32 genera consisting of the core microbiota. Approximately 91.6%±2.4% (mean ± SD) input 16S rDNA sequences can be positively assigned by the RDP Classifier to any phylum at a confidence threshold of 80%. However, under the same threshold, only approximately 38.9% (±4.8%) of 16S sequences can be assigned to a genus. Up to 61.1% of the 16S rDNA sequences cannot be positively assigned to any genus and remained unknown, suggesting that a substantial portion of the abomasal microbial diversity remained unexplored.

Species-level microbial composition in the bovine abomasal microbiota was analyzed using CD-HIT-OTU (Wu et al., 2011, submitted). A total of 90.3±2.9 OTU were identified in each abomasal microbial community. Seventy two OTU were present in the abomasal microbial communities of all 6 immune animals and likely represented the core microbiome of the abomasal microbial community. The community was dominated by 4 OTU, which accounted for approximately 50% of all 16S rDNA sequences in control animals. Among them, 2 OTU (OTU#1, 22.9% and OTU#7, 8.1%) were annotated to the genus *Prevotella* while the rest 2 OTU belonged to *Bacteroidales* and *Succinivibrio*. *Ostertagi ostertagi* infection significantly impacted only 2 OTU in immune animals (unadjusted *P* value<0.05). The relative abundance of OTU#32 (lineage: Catabacteriaceae) was increased from 0.4% in control to 1.0% in infected animals while the relative abundance of OTU#25 decreased from 0.3% in control to 0.1% in infected animals (*P*<0.05).

**Figure 6:**
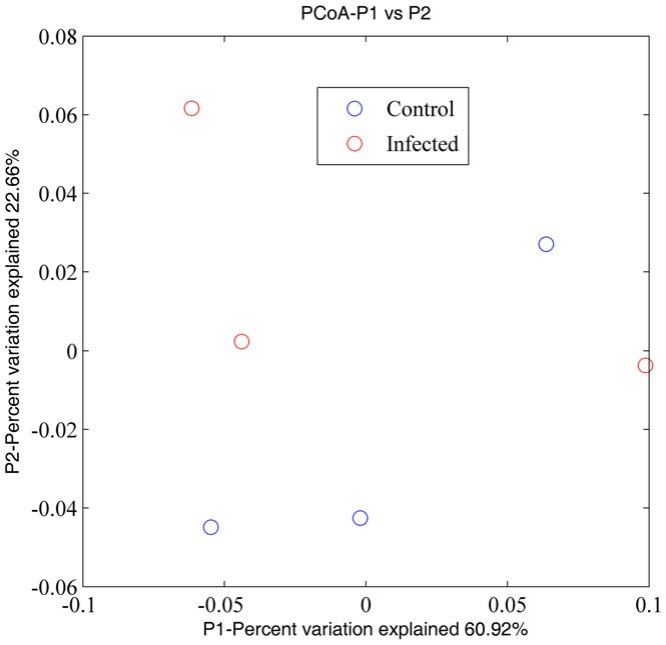
Principal Coordinates Analysis (PCoA) of weighted UniFrac values between control (blue) and infected (red) animals (*N* = 3): visualization of the first two dimensions (scaled).

**Figure 7:**
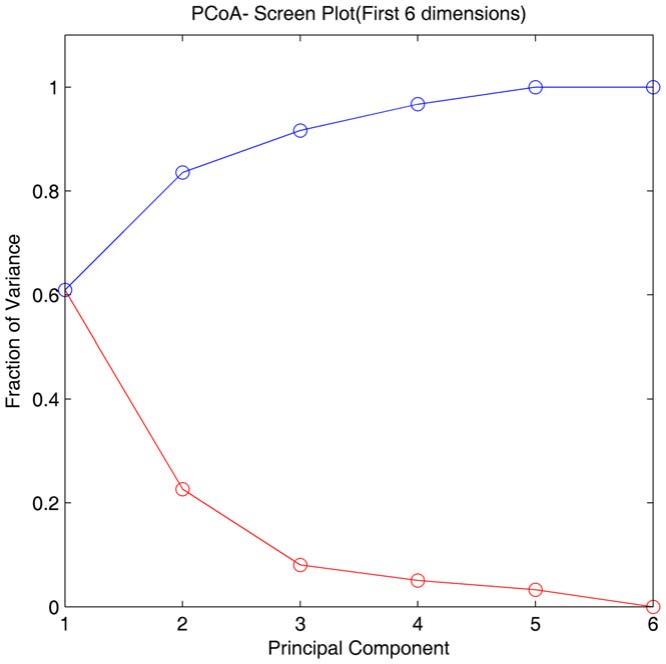
Principal Coordinates Analysis (PCoA) of weighted UniFrac values between control and infected animals (*N* = 3): The fraction of variance by the first six dimensions, both individually (red) and cumulatively (blue).

**Figure 8:**
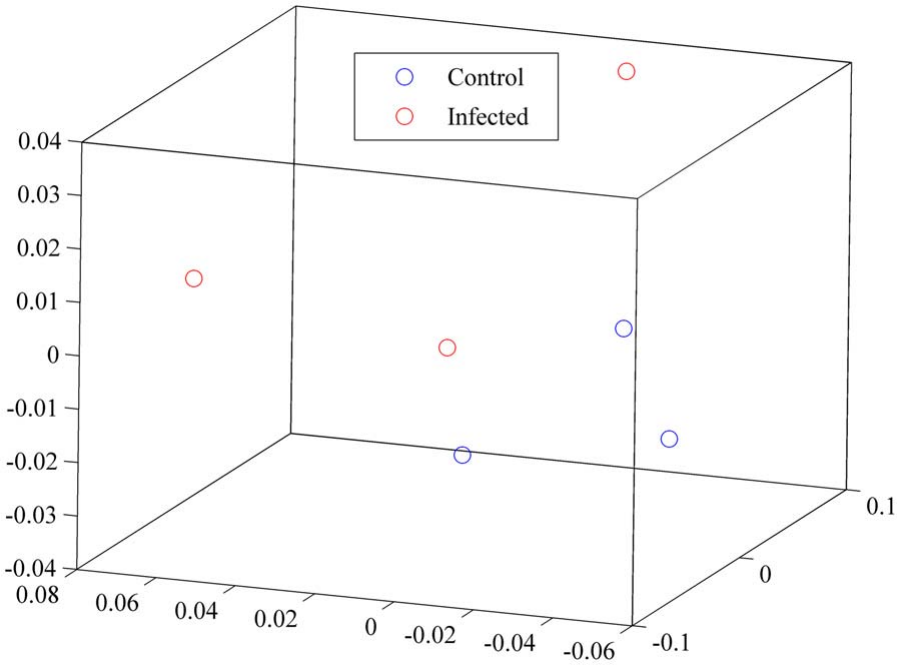
Principal Coordinates Analysis (PCoA) of weighted UniFrac values between control (blue) and infected (red) animals (*N* = 3): visualization in 3 dimensions (scaled).

Hierarchical clustering failed to group immune animals together in terms of their infection status based on relative abundances of the microorganisms making up the microbial community (Figure 5). Principal Coordinate Analysis (PCoA) provided further evidence that the separation between control and infected animals in their abomasal microbial composition derived from 16S rDNA sequences was not distinct (Figures 6, 7 and 8).

Richness indices ACE and Chao1 as well as Shannon-Weaver index were calculated at family-, genus-, and species (OTU)-levels in control and infected animals, respectively. These indices were not significantly different between two groups, suggesting that infection and ensuing abomasal pH elevation did not induce any notable change in biodiversity in the abomasal microbiota of immune cattle.

### Functional changes in the abomasal microbiota induced by infection

Functional and metabolic potentials of the bovine abomasal microbiota and possible alteration by parasitic infection were evaluated using WGS sequence reads. A total of 3,381 COG protein families were collectively identified in the bovine abomasal microbiota (Table S1). The most abundant COG families in the microbiota of control animals included signal transduction histidine kinase (COG#0642), ABC-type multidrug transport system (COG#1132), N_a_^+^-driven multidrug pump (COG#0534), DNA-directed RNA polymerase specialized sigma subunit, sigma24 homolog (COG#1595), and β-galactosidase/β-glucuronidase (COG# 3250). The most abundant COG functional categories (classes) in the abomasal microbiota of control animals (Table 2) were general function (Category R), translation, ribosomal structure and biogenesis (Cat. J), amino acid transport and metabolism (Cat. E), carbohydrate transport and metabolism (Cat. G), and replication, recombination and repair (Cat. L). Infection did not seem to have a significant impact on the function of the abomasal microbial community, and no significant difference in the number of hits assigned to any of 24 functional categories was detected between control and infected groups (Table 2).

**Table 2:** COG function classes identified in the bovine abomasal microbiota of immune animals.

COG Class	Description	Control	Infected	P value
R	General function prediction only	11.33±0.10	11.10±0.05	0.1375
J	Translation, ribosomal structure and biogenesis	8.47±0.04	8.44±0.16	0.8033
E	Amino acid transport and metabolism	8.31±0.10	8.43±0.09	0.4291
G	Carbohydrate transport and metabolism	8.23±0.15	8.91±0.16	0.0681
L	Replication, recombination and repair	8.09±0.18	7.75±0.10	0.2071
M	Cell wall/membrane/envelope biogenesis	7.86±0.08	7.90±0.15	0.7893
K	Transcription	5.81±0.04	5.76±0.10	0.6779
C	Energy production and conversion	5.33±0.13	5.39±0.01	0.6919
S	Function unknown	5.21±0.33	4.91±0.05	0.4559
T	Signal transduction mechanisms	4.41±0.30	4.59±0.02	0.6074
H	Coenzyme transport and metabolism	4.00±0.04	3.93±0.06	0.3883
O	Posttranslational modification, protein turnover, chaperones	3.81±0.09	3.77±0.01	0.7327
F	Nucleotide transport and metabolism	3.58±0.11	3.72±0.05	0.3488
P	Inorganic ion transport and metabolism	3.49±0.00	3.52±0.01	0.1791
I	Lipid transport and metabolism	3.06±0.04	3.06±0.05	0.9039
V	Defense mechanisms	3.02±0.03	3.02±0.02	0.8173
U	Intracellular trafficking, secretion, and vesicular transport	2.27±0.00	2.20±0.04	0.1931
D	Cell cycle control, cell division, chromosome partitioning	1.33±0.00	1.30±0.05	0.5934
Q	Secondary metabolites biosynthesis, transport and catabolism	1.19±0.02	1.15±0.09	0.7059
N	Cell motility	1.14±0.03	1.13±0.13	0.875
Z	Cytoskeleton	0.02±0.01	0.01±0.01	0.8461
B	Chromatin structure and dynamics	0.02±0.01	0.01±0.00	0.4023
A	RNA processing and modification	0.01±0.01	0.01±0.01	0.6511

*P* value was calculated based on unpaired *t*-test. The number denotes the percentage of hits annotated to a given functional class (mean ± SD; *N* = 3).

Predicted coding sequences (open reading frames or ORF) were also annotated against the Pfam database. Approximately 5,408 Pfam protein families, representing approximately 46% of all protein families in the database (Pfam v24.0), were identified from the metagenomic sequences obtained in the abomasal microbial communities with 4,604 and 4,587 families in control and infected groups, respectively. 3,783 Pfam protein families were common to both groups while 821 and 804 Pfam families were unique to control and infected groups, respectively. Neither the total numbers of Pfam protein families identified nor the number of unique protein families were statistically significant between the two groups, demonstrating a relative stability of the abomasal metagenomes in tolerating abomasal pH changes. The ABC transporter was most abundant protein family in the abomasum (Table 3), as in many other microbial communities, including in the bovine rumen [8]. Pfam protein families, PF00515 and PF07719, were among the 5 most abundant protein families in the abomasum (Table 3). Sixty-nine Pfam protein families, approximately 1.3% of Pfam families identified in the abomasal community, displayed a significant difference in abundance between control and infected groups at a false discovery rate (FDR)≤10%. Infection induced a significant increase in abundance in 27 Pfam protein families (data not shown). Most notable among these families were two related with bacterial two-component systems, response regulator receiver domain (PF00072), and a two component regulator propeller (PF07494). A total of 175 Pfam families with peptidase activities were identified in the metagenome dataset. None of these families were induced by infection and their abundance was not significantly changed in the infected group, compared to the uninfected control. Prolyl oligopeptidase family and peptidase families C10, S46 and M20 were among the most abundant peptidases in the abomasal microbial communities. Dipeptidyl peptidase (DPP) type IV (PF00930), a group of enzymes with important biological function including catalyzing a rate-limiting step in dietary protein digestion, was also abundant in the abomasum, possibly reflecting the predominance of the genus *Prevotella* in the abomasal microbiota.

**Table 3:** The 20 most abundant Pfam protein families in the bovine abomasal microbiota of immune animals.

Accession	Description	Control	Infected	P value	FDR
PF00005	ABC transporter	0.93±0.02	0.97±0.02	0.120	0.807
PF07719	Tetratricopeptide repeat	0.68±0.12	0.65±0.01	0.044	0.588
PF00004	ATPase family associated with various cellular activities	0.57±0.05	0.52±0.03	0.018	0.403
PF00515	Tetratricopeptide repeat	0.54±0.09	0.51±0.01	0.029	0.484
PF02518	Histidine kinase-, DNA gyrase B-, and HSP90-like ATPase	0.52±0.08	0.56±0.02	0.024	0.440
PF00501	AMP-binding enzyme	0.51±0.04	0.49±0.04	0.248	0.919
PF00593	TonB dependent receptor	0.49±0.07	0.48±0.01	0.867	1.000
PF01370	NAD dependent epimerase/dehydratase family	0.46±0.00	0.48±0.05	0.656	1.000
PF00535	Glycosyl transferase family 2	0.46±0.07	0.47±0.00	0.402	0.922
PF00155	Aminotransferase class I and II	0.43±0.02	0.45±0.00	0.646	1.000
PF00072	Response regulator receiver domain	0.42±0.03	0.48±0.00	0.001	0.089
PF07715	TonB-dependent Receptor Plug Domain	0.39±0.02	0.38±0.01	0.498	0.922
PF00873	AcrB/AcrD/AcrF family	0.38±0.02	0.42±0.01	0.067	0.653
PF00009	Elongation factor Tu GTP binding domain	0.37±0.05	0.41±0.02	0.064	0.637
PF07690	Major Facilitator Superfamily	0.37±0.02	0.41±0.00	0.048	0.588
PF03193	Protein of unknown function, DUF258	0.37±0.03	0.34±0.02	0.132	0.819
PF02321	Outer membrane efflux protein	0.35±0.02	0.32±0.01	0.048	0.588
PF07728	ATPase family associated with various cellular activities	0.35±0.00	0.32±0.01	0.109	0.772
PF00133	tRNA synthetases class I (I, L, M and V)	0.35±0.00	0.35±0.01	0.922	1.000
PF02463	RecF/RecN/SMC N terminal domain	0.35±0.03	0.35±0.01	0.460	0.922

*P* value and FDR were calculated using MetaStats. The number denotes the percentage of hits annotated to a given Pfam (mean ± SD; *N* = 3).

WGS sequences were further analyzed for Kyoto Encyclopedia of Genes and Genomes (KEGG) pathway assignment. Hits can be positively assigned to 300 KEGG pathways. Among them, ABC transporters pathway (Accession #02010) was the most abundant, representing 23.3% of all assignments in the abomasal microbiota of control animals, followed by two-component system (#02020) at 8.9%, purine metabolism (#00230) at 2.0%, pyrimidine metabolism (#00240) at 1.4%, and amino sugar and nucleotide sugar metabolism (#00520) at 1.3%. Infection did not seem to induce any significant changes in abundance in the 20 most abundant KEGG pathways. Select pathways significantly impacted by infection are listed in Table 4. The number of hits assigned to bacterial invasion of epithelial cells (#05100) and gastric acid secretion (#04971) pathways were seemingly reduced by infection. On the other hand, the number of hits assigned to nitrogen metabolism (#00910), cyanoamino acid metabolism (#00460), and α-linolenic acid metabolism (#00592) were probably increased by infection.

**Table 4:** KEGG pathways impacted by *Ostertagia ostertagi* infection in immune animals.

Accession	KEGG Pathway Description	Control*	Infected*	P value**
#05100	Bacterial invasion of epithelial cells	0.084±0.002	0.059±0.000	0.0033
#00910	Nitrogen metabolism	0.523±0.001	0.553±0.002	0.0044
#00460	Cyanoamino acid metabolism	0.277±0.002	0.311±0.002	0.0056
#00622	Xylene degradation	0.099±0.000	0.094±0.000	0.0078
#00592	α-Linolenic acid metabolism	0.052±0.001	0.045±0.000	0.0090
#04971	Gastric acid secretion	0.204±0.003	0.178±0.002	0.0100

Note:

*the percentage (mean ± SD) of hits annotated to a given pathway.

**un-adjusted *P* value was calculated using unpaired *t*-test.

## Discussion

In an attempt to dissect mechanisms underlying protective immune responses to *Ostertagia ostertagi* infections in cattle, which develops very slowly and requires a prolonged exposure before becoming effective, we developed partially immune animals using multiple drug-attenuated infections. While host mechanisms underlying the development of long-term protective immunity have recently been discussed [4], the gut microbiota of ruminants has not been systematically characterized until recently [8]–[10]. Three-way interactions between the host, its microbiota and parasites are little understood. In this study, we characterized the bovine abomasal microbiota using metagenomic tools. Our results provided the first piece of evidence that a minimal disruption in the bovine abomasal microbiota by the parasitic nematode may contribute equally to the restoration of gastric function in immune animals.

The abomasum is an important yet unique organ. Its low luminal pH environment, normally ranging from pH 1 to 3, is essential to activation of digestive enzymes and absorption of nutrients. The abomasal acidity is also a critical determinant in the pathogenesis of many diseases, including abomasal ulceration, abomasitis, abomasal bloat, and gastric tumors. While this extreme acidic environment serves as a potent barrier against bacterial infection and functions as an abomasal sterilizer, many enteric microorganisms, such as *Escherichia coli* and *Salmonella typhimurium*, have evolved elegant mechanisms to cope with the potentially lethal effects of acid stress that allows them to tolerate drastic pH fluctuations in their ever-changing surroundings and during pathogenesis [11]–[12]. Cataloguing biodiversity in this environment will facilitate our understanding of survival strategies of microorganisms under stress, which could have important implications in animal and human health. *Ostertagia ostertagi* infections did not seem to impact the abomasal microbial diversity in immune cattle. The number of genera identified from control and infected groups was not statistically different. Only 32 genera were identified in all samples tested, possibly representing the core microbiome of the bovine abomasal microbial community (Figure 5).

*Prevotella* is among the most abundant genera from the rumen and hindgut of ruminants, functioning mainly in the breakdown of protein and carbohydrate substrates. It is conceivable that peptidases secreted by *Prevotella* species may play an important role in protein digestion in the bovine gut. Compared to phylogenetic profiles of the human stomach microbiota obtained using 16s rDNA sequencing [13], the bovine abomasal microbiota compositions displayed a unique pattern. The human stomach microbiota is dominated by 5 major phyla: Proteobacteria, Firmicutes, Bacteroidetes, Actinobacteria, and Fusobacteria, which is significantly different from the microbial communities in other human organs, suggesting the human stomach harbors a distinct microbial ecosystem. Recent studies suggest that the number of microorganisms in the human stomach is related to luminal pH changes induced by diseases and therapy [14]–[15].

The gastrointestinal tract is the organ with the richest proteases (peptidases) of both endogenous and exogenous origin. Protein degradation in ruminants is controlled by microorganisms and is strongly influenced by gut luminal pH [16]. In the rumen, intact dietary proteins are first converted to shorter oligopeptides by proteases. The resultant oligopeptides are degraded via a biphasic mechanism: oligopeptides are cleaved first to dipeptides and/or tripeptides by DPP, which are further degraded by di- or tri-peptidases to amino acids [16]. Peptide hydrolysis in the human intestine seems to follow the same biphasic process [16]. Protein requirements for ruminants include both dietary protein that escapes ruminal degradation (i.e., ruminally undegraded protein or RUP) and ruminally-synthesized microbial protein reaching the duodenum. Dairy cows require a proper balance of RUP and ruminally degraded protein (RDP) in their diets to best meet nutritional requirements for metabolizable protein and achieve desired nitrogen efficiency. Proteases of microbial origin in the rumen and hindgut play an important role in ruminant nutrition as well. In addition, proteases are able to serve as signaling molecules. Exogenous proteases can activate protease-activated receptors (PAR) in host enterocytes, thereby providing a novel mechanism of the interactions between gut microbiota and host cells [17]. Understanding gut protease balance and its regulation represents an important aspect in dissecting the pathophysiology of gastrointestinal diseases. A total of 175 Pfam families with peptidase activities were identified in our metagenome datasets, including prolyl oligopeptidase family, peptidase families C10, S46 and M20 as well as DPP type IV (PF00930). None of these families seemed induced by infection and their abundance remained stable upon reinfection. However, a KEGG pathway on nitrogen metabolism (Acc# 00910) was impacted by infection in immune animals. Nevertheless, it is conceivable that pH-induced changes in the abomasal microbial community composition could have an important impact on host nutrition and physiology. Infection and ensuing elevation of the abomasal pH in immune animals did not appear to affect the abomasal proteolytic capability, suggesting that the development of protective immunity in immune animals may include mechanisms in regulating their gut microbiota and its secretory capabilities.

Two-component regulatory systems (2CRS) enable microorganisms to sense, respond, and adapt to a wide range of adverse environmental conditions. A membrane-bound histidine kinase functions as a sensor and transmits detected stimuli/signals to a corresponding response regulator to mediate the cellular response. This generally leads to differential expression of target genes, resulting in adaption or stress tolerance or even cross-protection responses [18]. Many of the two-component systems are involved in signaling systems for sensing the changes of external environment such as temperature, osmolarity, chemo attractants, and pH. The latter regulates genes in many biological processes, including amino acid and sugar catabolism, electron transport, oxidative stress, and periplasmic and envelope proteins in *E. coli* [19]. While many enterobacteria prefer to grow in neutral pH environments, they nevertheless experience dramatic pH fluctuations in their native habitats [11], especially after feeding cycles. On the other hand, uncontrolled metabolic activities of enterobacteria also induce drastic pH changes in their own habitat. For example, rapid fermentation of starch by *Streptococcus bovis* in the rumen often leads to an increase in ruminal lactate production and results in a decrease in ruminal pH, which in turn promotes its own proliferation, creating a feedback loop and contributing to the progress of rumen acidosis and many other pathological conditions. As a result, it is often attempted to control *S. bovis* growth in the gut microbial ecosystem. Recently a two-component signal transduction system involving the control of *S. bovis* growth has been identified [20]. *Streptococcus* species were indeed present in the abomasal microbial communities of both control and infected animals; and the two-component system was the second most abundant pathways in these communities. Our data showed that infection induced a significant increase of two 2CRS-related protein families, two component regulator propeller (PF07494) and response regulator receiver domain (PF00072). Our results suggest that the abomasal pH elevation may elicit activation of 2CRS, which in turn promoted survival or even proliferation of acid-tolerant species in the gut of infected animals. Ecophysiological implications of pH-induced changes in the microbiota composition remain largely unknown. However, a slightly elevated pH would allow proliferation of certain acid-tolerant species, such as *Streptococcus bovis*, which has been shown to produce an inhibitor of gastrin secretion by the host cell [21], modulating systemic host responses.

Host-parasite interactions in the cattle-*Ostertagia* system have been well understood. Parasites display their ability to impact host normal physiology by hijacking their proteolytic pathways via structural mimicry [22]–[23]. Most notably, parasites are able to modulate acid secretion and pepsinogen production of the host, resulting in a systemic response. During a primary *Ostertagia ostertagi* infection in cattle, the acidity of the abomasal content decreases markedly to pH 5 to 6, leading to hypergastrinamia. Similarly, *Ostertagia leptospicularis* infection in sheep is able to mediate inhibition of abomasal acid secretion [24], suggesting the parasites have abilities to induce changes in their environment that favors their survival and increase their reproduction. Indeed, the gastric acid secretion pathway was significantly impacted by infection (Table 4). On the other hand, the host cell is able to trigger a cascade of events, including activation of the complement system and recruitment of leukocytes to the site of infection. The amplification loop resulting from activation of the alternative pathway could allow for a sustained elevation of inflammatory cytokines. This, along with reactive oxygen species and nitride oxide as well as proteases released by infiltrates, leads to amplification of local inflammation, thus creating a hostile environment for developing worms and providing an efficient containment for parasites [4], [25]. In addition, the host cell has abilities to enhance tissue repair and promote its mucin-secreting function. These factors are instrumental in the development of protective immunity.

The host and its microbiota have developed a mutualistic relationship during evolution. Normal gut microflora is essential for the host function. The abomasal microbiota harbors bacteria that produce a potent inhibitor of gastrin secretion in the host [21], resulting in the reduction of gastrin levels by approximately 90%, therefore directly impacting host physiology. The ruminal fluids from the same animals display a similar inhibition of gastrin secretion, suggesting the gastrin inhibitors might be produced by rumen microorganisms that survive in the abomasum, which may account for the bulk of observed increases in anaerobic populations in the abomasal ecosystem when the luminal pH is elevated to 3.5 or above. Gastrin inhibitory activities is pH-dependent, suggesting normal pH environment in the abomasum may not favor production of the inhibitor. Our results indicated that the abomasal microbiota can produce an abundant level of serpins, a large class of protease inhibitors involved in regulation of a wide spectrum of physiological processes. Many gut bacteria, especially those commensal bacteria, are facing constant attack in their native habitat by proteases secreted by host cells, such as neutrophils [26]. Serpins produced by the abomasal microbiota prevent attachment of host proteases, playing an important role in the interaction between the abomasal microbiota and the host. In addition, it has been known that intestinal parasite infection tends to increase dietary lysine requirements by up to 50% [27]. One possible mechanism is that parasite infection alters gut microbiota composition and thus reduces the production of essential amino acids by the microbiota. Our results suggest that unlike in naive animals, *Ostertagia* infection in immune cattle induced a minimal disruption in the abomasal microbiota, which may contribute equally to the development of long-term protective immunity. While immune animals may develop abilities to maintain proper stability of their abomasal microbiota, metagenome plasticity in the bovine abomasum was evident. Approximately 1.3% of Pfam protein families identified in the microbial communities displayed a significant difference in abundance upon reinfection. The microbiota was responsive to external changes in the environment, which allows certain species to fluctuate in the population, to explore newly available niche space, and yet maintain overall function of the entire community by expressing a stable level of various proteins. It is foreseeable that a holistic understanding of the complex three-way interactions between the host, its microflora, and parasites will lead to the improvement of animal and human health.

## Materials and Methods

### Animals and Parasitology

Animals and standard parasitology protocols were previously described [4]. Briefly, Holstein bull calves obtained shortly after birth were maintained on concrete throughout the experiment and fed *ad lib* a standard calf ration after weaning. All animal work has been conducted according to Institutional Animal Care and Use Committees guidelines and approved by the Beltsville United States Department of Agriculture - Agricultural Research Service (USDA-ARS) Animal Care and Use Committee (Approval ID: lcg#05-013). Oral infection of *Ostertagia ostertagi* infective L3 larvae (10^5^ larvae/calf) was initiated after calves reached 3–4 months of age. All 6 calves were initially orally infected with a single dose of 10^5^ L3 infective larvae for 14 days and then treated with a 2× labeled dose of fenbendazole to remove existing parasites. The calves were allowed to rest for 30 days on concrete before a 2^nd^ round of infection with the same number of L3 larvae for 14 days. This infection-drug treatment-resting cycle was repeated 4 times on all calves used. These calves became partially immune after 4 drug-attenuated infections based on pre-defined parasitological and immunological parameters, including a significant reduction in worm burden, an increase in the percentage of larvae and a change in cytokine expression profile. During the final drug-attenuated infection, three calves were drug treated and allowed to rest for 3–4 weeks and then orally dosed with a tap water placebo. These calves were used as controls. The remaining three calves, which also underwent 4 rounds of infection-treatment-resting procedures and allowed to rest 3–4 weeks between the treatments, were orally infected with a single-dose of 10^5^ L3 infective larvae for 14 days. At the end of the experiment, calves were sacrificed, and the abomasal contents were collected. The abomasal luminal pH was measured using a standard pH meter. The sample was snap frozen in liquid nitrogen prior to storage at −80°C until DNA was extracted. Fecal egg count (EPG) was monitored during the repeat infection experiment using zinc sulfate double centrifugation, and parasite burdens were determined as previously described [28].

### Metagenomic DNA extraction and amplicon preparation

Metagenomic DNA was extracted from abomasal contents using a QIAamp DNA stool kit (Qiagen, Valenica, CA) with some modifications. A 5-min incubation at 95°C was used to replace the 70°C lysis recommended in the standard protocol. DNA integrity was verified using a Bioanalyzer 2000 (Agilent, Palo Alto, CA). DNA concentration was quantified using a QuantiFluor fluorometer (Promega, Madison, WI). A 570-bp region of the 16S rRNA gene (*E. coli* position 357 to 926) containing hypervariable regions V3– V5 of the 16S rDNA was amplified from 40 ng of metagenomic DNA with 8-bp sample-specific barcoded primers using 2.5 units of AccuPrime Taq DNA Polymerase High Fidelity (Invitrogen, Carlsbad, CA) in a 50-µl reaction buffer containing 200 nM primers, 200 nM dNTP, 60 mM Tris-SO_4_, 18 mM (NH4)_2_SO_4_, 2.0 mM MgSO4, 1% glycerol, and 100 ng/ul bovine serum albumin (New England Biolabs, Ipswich, MA). These regions were selected for analysis because of their high variability [29]. PCR was performed using the following cycling profile: Initial denaturing at 95°C for 2 minutes followed by 25 cycles of 95°C 30 s, 50°C 30 s, and 72°C 120 s. The amplicons were generated from each metagenomic DNA sample separately, purified using a Agencourt AMPure XP kit (Beckman Coulter Genomics, Danvers, MA), and quantified using a QuantiFluor fluorometer. The amplicons from individual samples were pooled in equal mass (molar) ratios. The amplicon pool at the desired size (∼672 bp including primers and adaptors) was excised from 1.0% agarose gel and purified using a QIAquick Gel Extraction Kit (Qiagen). The purified amplicon library was further verified and quantified using a BioAnalyzer 2000 (Agilent) and subject to Roche/454 pyrosequencing.

### Roche/454 Pyrosequencing

The abomasal microbiota was characterized by two sequencing approaches using the Roche/454 GS FLX Titanium chemistry, the 16S rRNA gene (hypervariable V3–V5 regions) and the whole genome shotgun (WGS). For the first approach, unidirectional sequencing of amplicon libraries was performed according to the manufacturer's instructions with a modification (App No 001-2009, Roche Applied Science, Indianapolis, IN). This modification, using a specific fusion primer design, accommodates amplification using the GS FLX Titanium emPCR Kits (Lib-L). Five hundred ng of DNA were used to generate libraries using the GS FLX Titanium Rapid Library Preparation method for WGS sequencing. Therefore, emulsion PCR (emPCR) was carried out using a Lib-L kit for both approaches. Pyrosequencing was conducted using a GS FLX Titanium System (Roche) following the manufacturer's protocol.

### Sequence analysis, protein prediction and annotation

16S rDNA raw sequence reads were first decoded based on sample-specific 8-bp bar codes; their quality was checked, and artifacts were removed. Sequence reads shorter than 200 bp were excluded. The sequence read that passed the quality filters (41417±23932.8 per sample) were analyzed using the RDP classifier [30] at both 80% and 95% confidence threshold levels for taxonomic classification and phylogenetic inference.

The 16S rDNA sequences were then analyzed using CD-HIT-OTU (Wu et al., 2011, submitted) for the abomasal microbial composition at the species (OTU) level. This algorithm uses a greedy incremental clustering process to identify OTU from 16S rDNA tags, which involves 3 major steps: raw read filtering and trimming, selection of error-free reads, and clustering selected representative reads into individual OTU at a user-specific cutoff (97% identity in this case for species level clustering). The program avoids over estimation of OTU, a common problem for many existing programs, and results in a rapid and more accurate estimation of microbial diversity in complex microbial ecosystems. OTU identified were then annotated using FR-HIT [31] against the GreenGene database.

The 16S rDNA sequences were further analyzed using Fast UniFrac [32]. Briefly, the core set of the 16S GreenGene database was downloaded, and the input 16S sequences were analyzed using MegaBLAST. The resultant hit table was input into the Fast UniFrac server for Principal Coordinates Analysis (PCoA).

Several quality control filters were applied to WGS raw reads before analysis. First, host contaminants (sequences of bovine origin) were removed using FR-HIT [31] against the bovine genome Btau 4.0 (cutoff: 95% identity over 95% of the entire sequence length). The possible artifacts (redundant reads) were then identified and excluded using CD-Hit [33]. Quality WGS sequences were *de novo* assembled using the Newbler software (v2.5.3). Coding sequences (CDS or ORF) were predicted from all contigs and singletons ≥300 bp using FragGeneScan (v1.14), a recently developed program combining sequencing error models and codon usages in a hidden Markov model to improve the prediction of protein-coding region in short reads [34]. CDS were also predicted in parallel using Metagene [35]. Functional annotation was performed according to the COG, KEGG, and Pfam (v24.0) databases. Profile HMMs of Pfam 24.0 were downloaded, and Pfam annotation was conducted using the HMMER software package, version 3.0rc2 (downloaded from ftp://selab.janelia.org/pub/software/hmmer3/). Post-filtered sequence reads were analyzed using BLAST (v2.2.21) at an E-value cutoff 1.0E-05 against the NCBI NR database (downloaded 1/21/2011). Statistical analysis was carried out using an online version of MetaStats [36].

## Supporting Information

Table S1 (XLS)
